# Supplementary material for: Highly efficient construction of monkey blastoid capsules from aged somatic cells
Source: Nat Commun. 2025 Jan 28;16:1130. doi: 10.1038/s41467-025-56447-z (PMC11775175; doi:10.1038/s41467-025-56447-z)
Supplement: Supplementary file 1 — Supplementary Information [file 41467_2025_56447_MOESM1_ESM.pdf]

Supplementary Fig. 1

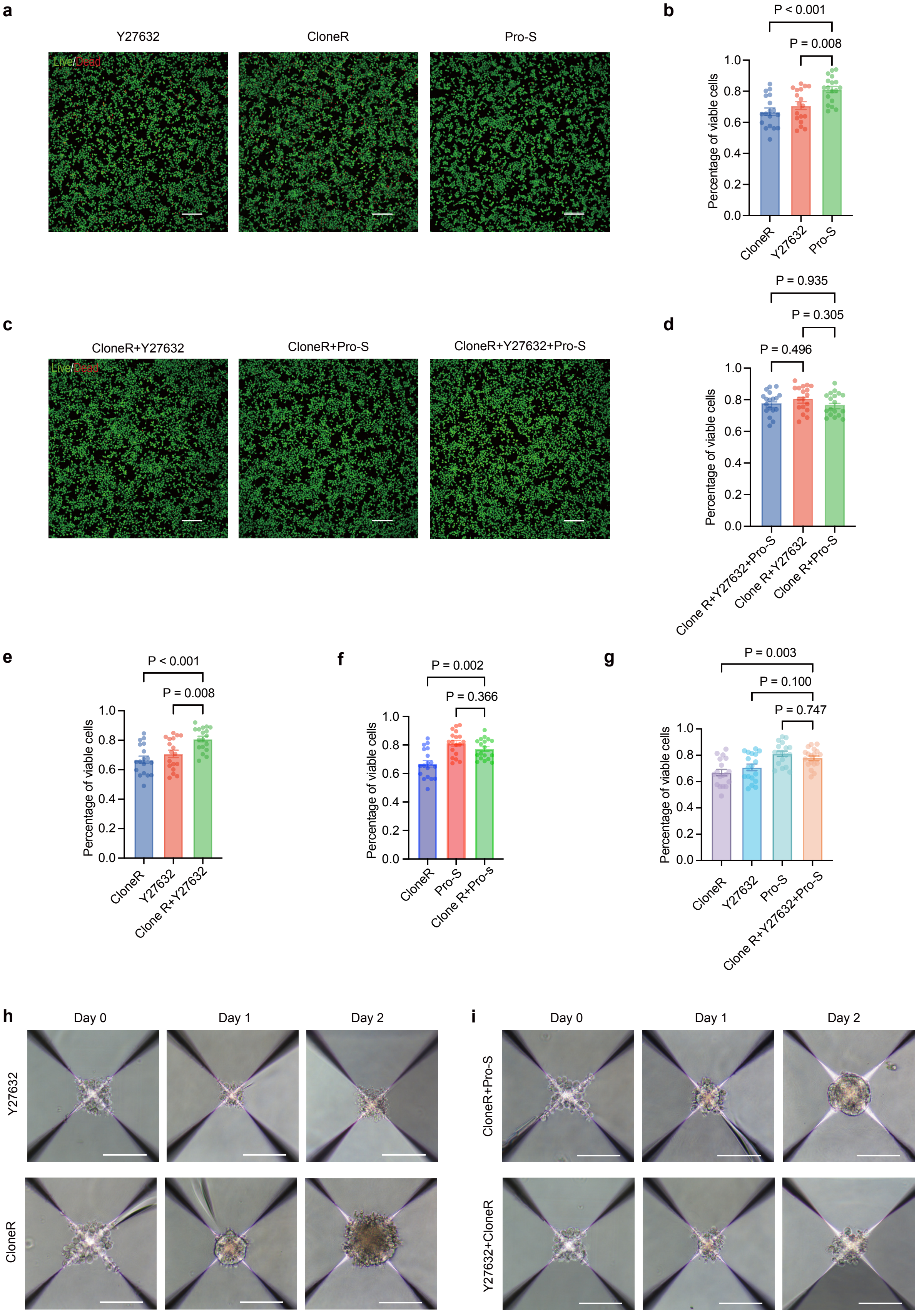

Supplementary Fig.1. The effect of Pro-S on cell viability.

a Representative immunofluorescence images of live (green)/ dead (red) cells of Y27632, CloneR and Pro-S groups. Scale bars, 200 µm. **b** Viable cell ratio obtained from the live/dead staining assay of Supplementary Fig.1a. n = 18 biological replicates, ordinary one-way ANOVA test; Error bars, mean ± S.E.M. **c** Representative immunofluorescence images of live (green)/ dead (red) cells of CloneR+Y27632, CloneR+Pro-S, CloneR+Y27632+Pro-S groups. Scale bars, 200 µm. **d** Viable cell ratio obtained from the live/dead staining assay of Supplementary Fig.1c. n = 18 biological replicates, ordinary one-way ANOVA test; Error bars, mean ± S.E.M. **e-g** Viable cell ratio obtained from the live/dead staining assay of Y27632, CloneR, Pro-S and different combination; n = 18 biological replicates, ordinary one-way ANOVA test; Error bars, mean ± S.E.M. **h** Representative phase-contrast images show the effect of Y27632, CloneR on blastoids formation efficiency. Scale bars, 200 µm. **i** Representative phase-contrast images show the effect of CloneR+Y27632, CloneR+Pro-S on blastoids formation efficiency. Scale bars, 200 µm.

Supplementary Fig. 2

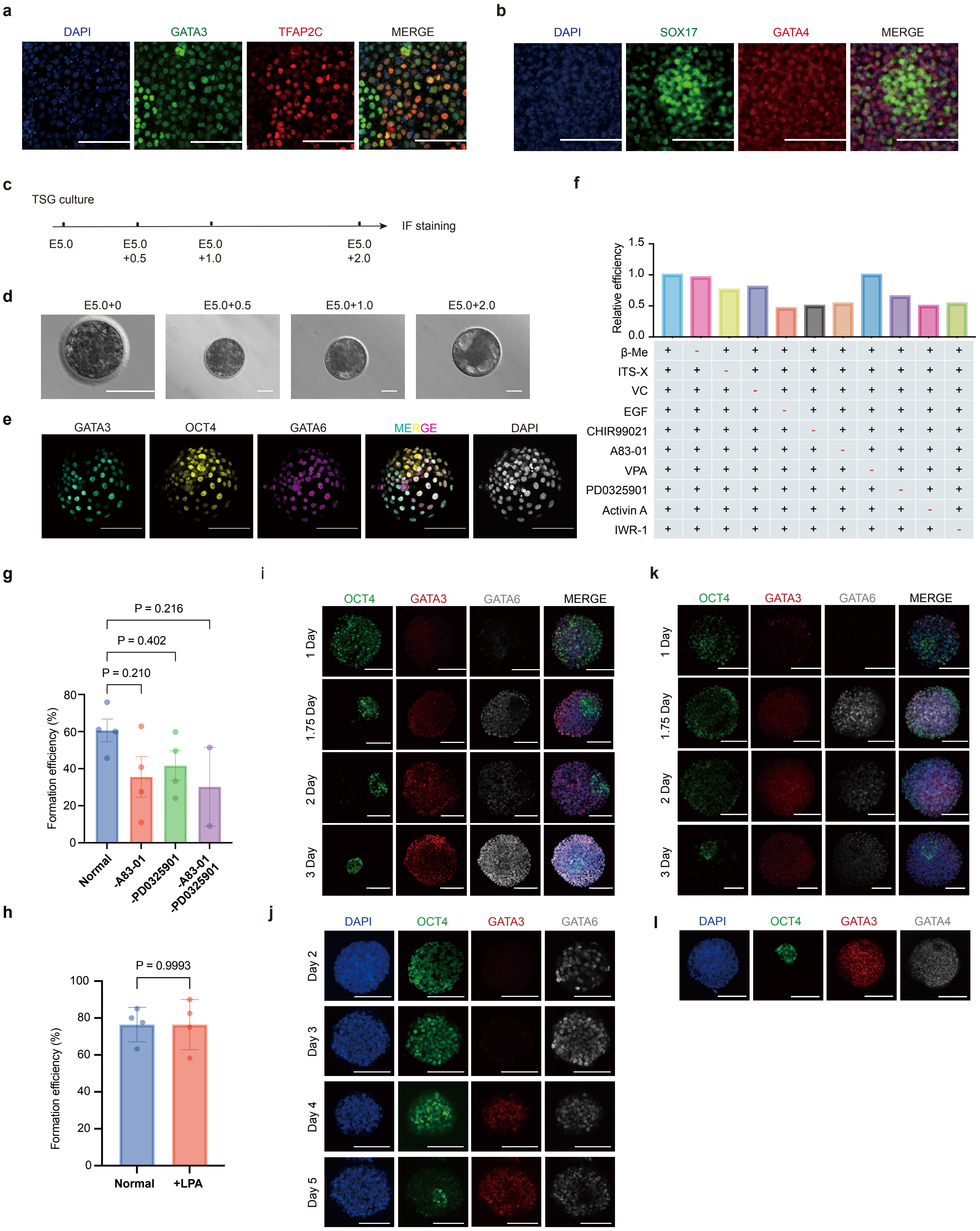

Supplementary Fig.2. Cell-lineage during monkey blastoid formation.

a Immunofluorescence staining images of GATA3, TFAP2C in monkey TLCs derived from M-ESCs. Scale bars, 100 µm. **b** Immunofluorescence staining for SOX17, GATA4 in monkey HLCs derived from M-ESCs. Scale bars, 100 µm. **c** Schematic depiction of monkey embryos-E5.0 cultured with TSG. **d** Representative bright field images of monkey embryos-E5. Scale bars, 200 µm. **e** Representative immunofluorescent staining images of GATA3/OCT4/GATA6 in monkey embryos-E5+2 days. Scale bars, 100 µm. **f** The effect of each TSG component on cavity formation efficiency. **g** Cavity formation efficiency of monkey blastoids cultured without A83-01, PD0325901, A83-01 and PD0325901 compared with complete TSG. n = 4 technical replicates, Ordinary one-way ANOVA test; Error bars, mean ± S.E.M. **h** Cavity formation efficiency of monkey blastoids cultured with TSG compared with additional LPA. n = 4 technical replicates, Error bars, mean ± SD. unpaired two-tailed *t*-test. **i-j** Representative immunofluorescence co-staining images of OCT4, GATA6 and GATA3 in cell aggregates generated using 1E5 (i) and 2E5 (j) at higher passage ESC starting cell numbers and HDM culture time. Scale bars, 100 µm. **k** Representative cell aggregates co-stained for GATA3, OCT4 and GATA4 visualized by immunofluorescence. Scale bars, 200 µm. **l** Representative immunofluorescence co-staining of OCT4, GATA6 and GATA3 in cell aggregates generated at different time points during blastoid formation. Scale bars, 200 µm.

Supplementary Fig. 3

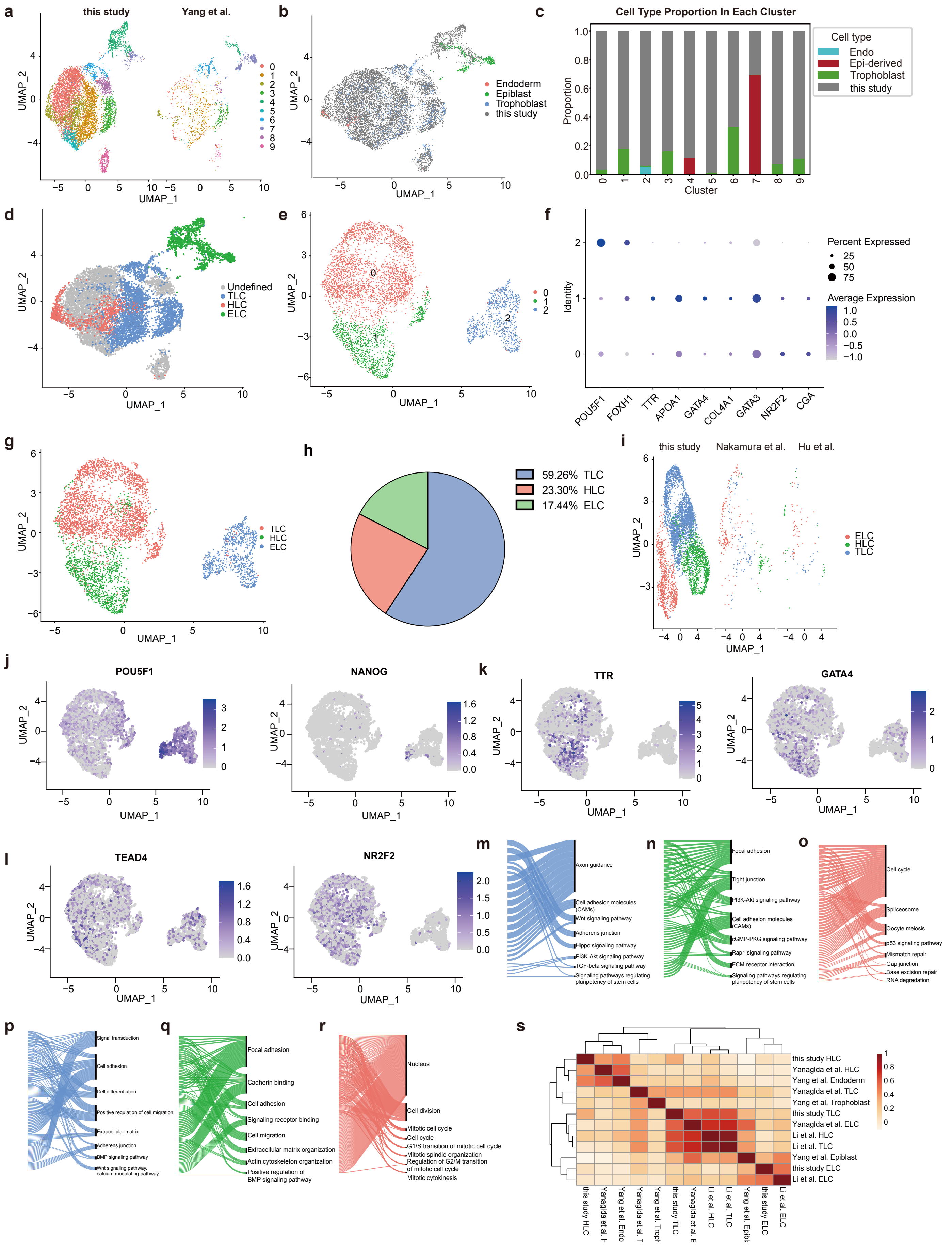

Supplementary Fig.3. Single-cell transcriptional profiling of monkey blastoids.

a UMAP of single-cell transcriptomes of cells from monkey blastoids, and monkey natural embryos. **b** UMAP of monkey natural embryos and monkey blastoids scRNA-seq comparison. **c** Proportion of cells in each cluster derived from blastoids. **d** UMAP plot of monkey blastoid cells integrated with published monkey embryo cells. Undifined cells were in grey. **e** UMAP plot showing that monkey blastoids included three major lineages, excluding undefined cells. **f** Identification of cell types based on lineage-specific markers of blastocysts. **g** 0, 1, 2 subclusters were identified as ELC, HLC and TLC respectively based on lineage markers. Individual cells are colored by origin: TLC (red), HLC (green), ELC (blue). **h** Pie chart showing the frequencies of ELC, TLC, and HLC in monkey blastoids indicated by lineage markers. **i** UMAP of single-cell transcriptomes of monkey blastoids, published monkey preimplantation embryos. **j-l** Projection of lineage-specific markers on the UMAP revealed the distribution of major blastoid clusters. **m-o** KEGG enrichment of high-expressed genes in ELC (m), HLC (n) and TLC (o). **p-r** Gene oncology enrichment of high-expressed genes in ELC (p), HLC (q) and TLC (r). **s** Correlation analysis of blastoid clusters with related datasets.

Supplementary Fig. 4

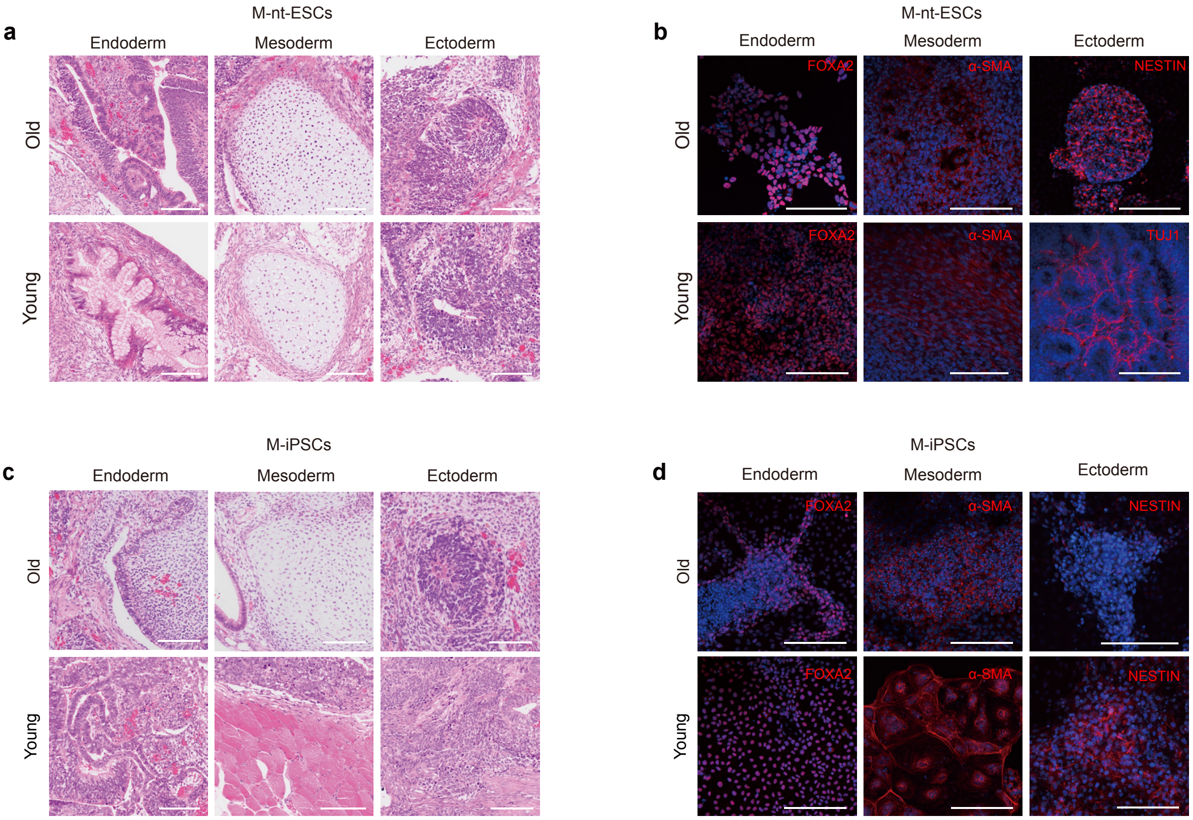

Supplementary Fig.4. Further characterization of M-nt-ESCs and M-iPSCs. **a** Representative images showing histological analysis of teratomas generated from M-nt-ESCs with old and young origins. Scale bars, 50 µm. **b** Representative immunofluorescence images showing *in vitro* EB differentiation of M-nt-ESCs with old and young monkey origins. Scale bars, 200 µm. The pseudo-colors were used. **c** Representative images showing histological analysis of teratomas generated from M-iPSCs with old and young origins. Scale bars, 50 µm. **d** Representative immunofluorescence images showing *in vitro* EB differentiation of M-iPSCs with old and young monkey origins. Scale bars, 200 µm. The pseudo-colors were used.

Supplementary Fig. 5

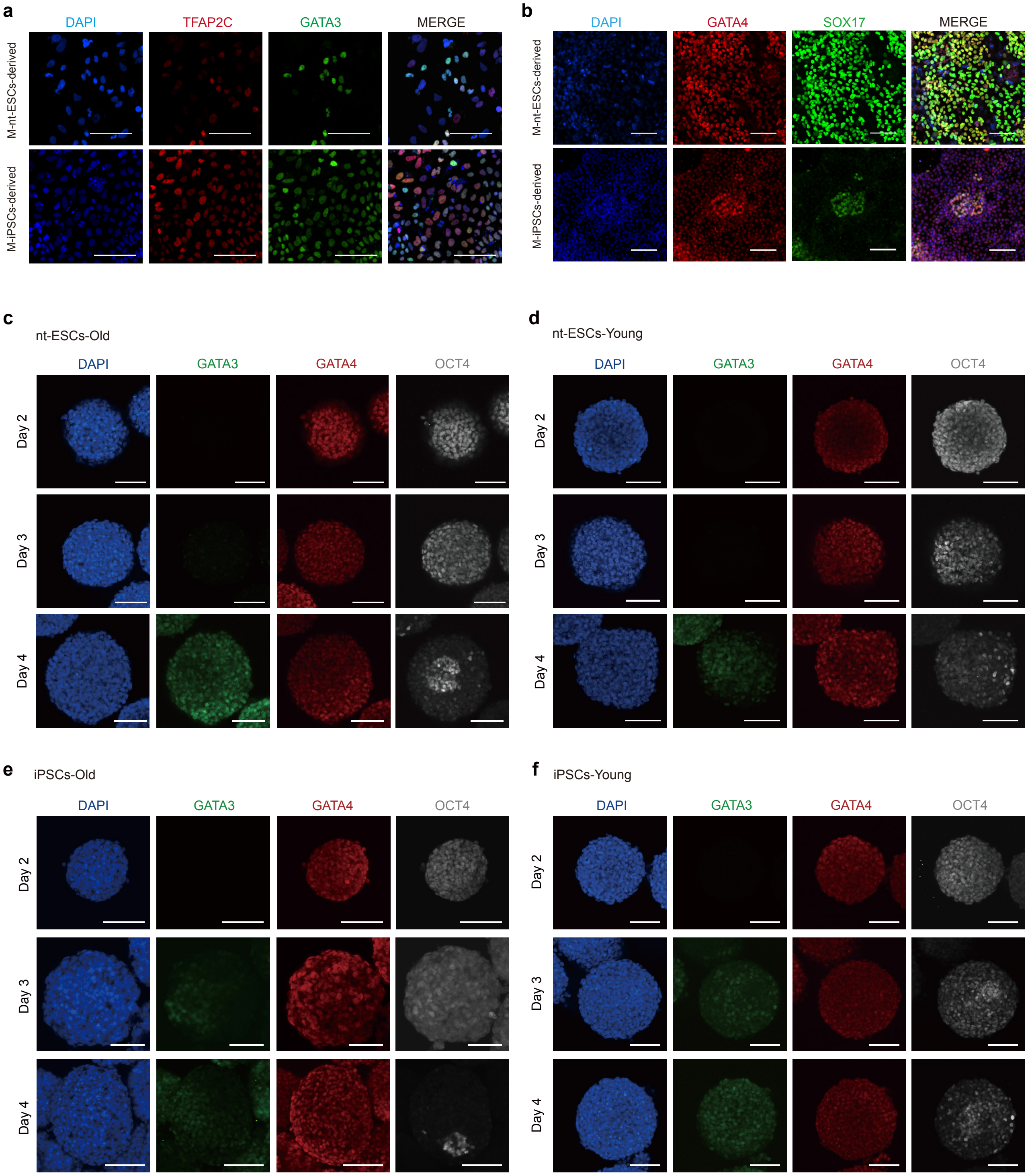

Supplementary Fig.5. Cell-lineage during monkey blastoid formation from M-nt-ESCs and M-iPSCs.

a Representative immunofluorescent staining of TFAP2C/GATA3 showing differentiation of monkey M-nt-ESCs (top) and M-iPSCs (bottom) into trophectoderm lineage. Scale bars, 100 µm. **b** Representative immunofluorescent staining of GATA4/SOX17 showing differentiation of monkey M-nt-ESCs (top) and M-iPSCs (bottom) into hypoblast lineage. Scale bars, 100 µm. **c-f** Representative immunofluorescence co-staining of OCT4, GATA4 and GATA3 in cell aggregates generated at different time points during blastoid formation from old (c)- and young (d)- M-nt-ESCs, old (e)- and young (f)- M-iPSCs. Scale bars, 100 µm.

Supplementary Fig. 6

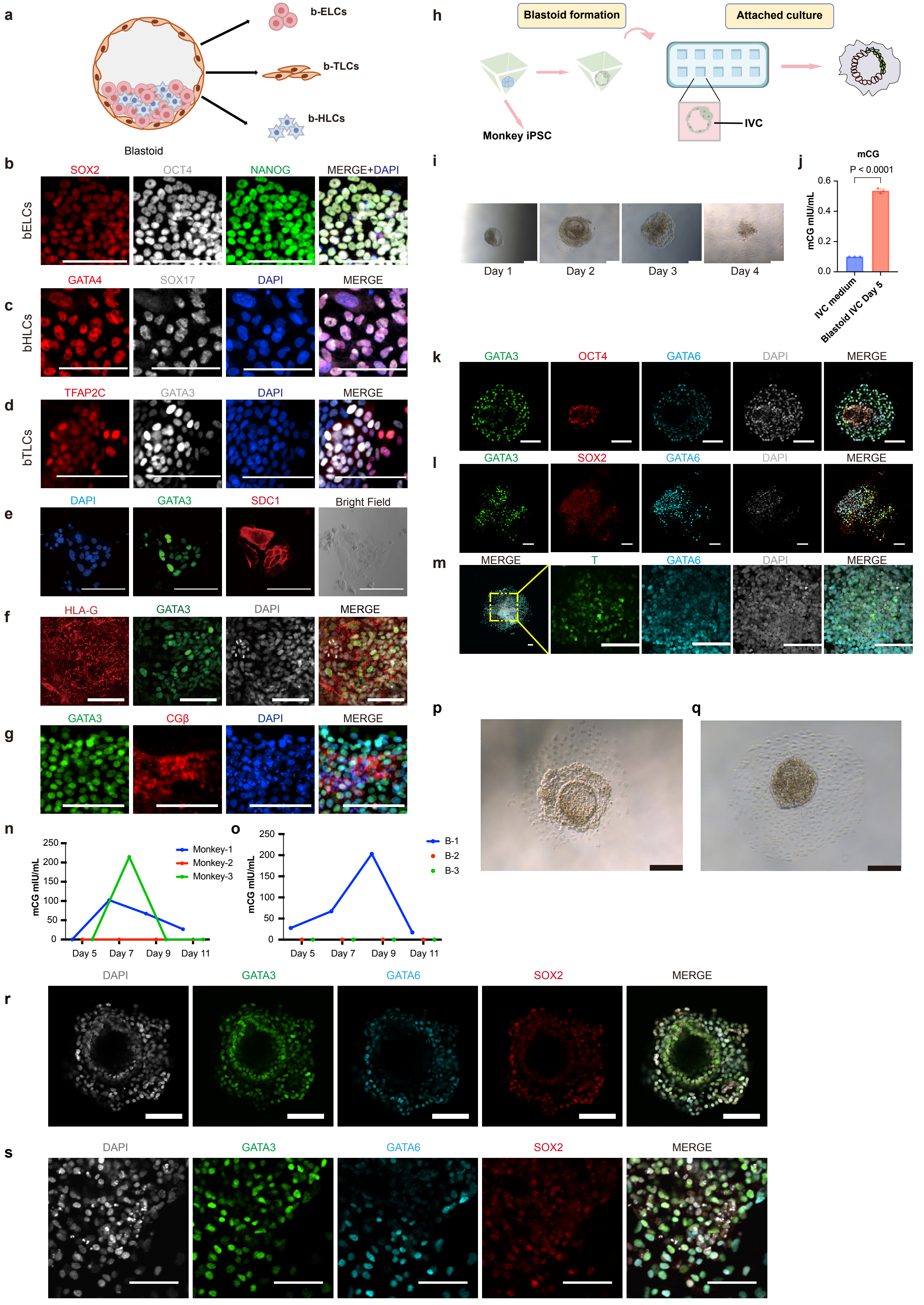

Supplementary Fig.6. Lineage stem cells derivation and *in vitro* culture of monkey blastoids

a Schematic diagram of the derivation of b-ELCs, b-TLCs and b-HLCs cells from blastoids. **b-d** Representative immunofluorescence images of b-ELCs(b), b-HLCs(c) and b-TLCs(d). Scale bars, 100 µm. **e-g** Representative immunofluorescent staining images of marker genes detection in STB-like cells (SDC1) (e) and EVT-like cells (HLA-G) (f) and (CGβ) (g). Scale bars, 100 µm. **h** Schematic diagram shows the process of blastoid *in vitro* culture. **i** Representative bright field images of monkey blastoid IVC on day 1-4. Scale bars, 200 µm. **j** mCG in blastoid IVC on day 5 compared with IVC medium as control. n = 3 technical replicates, Error bars, mean ± SD. unpaired two-tailed *t*-test. **k-m** Representative immunofluorescent staining images of monkey blastoid IVC on day 2 (k), day 4 (l) and day 5 (m). Scale bars, 100 µm. **n** The level of mCG after natural blastocysts transplantation from day 5 to day 11. n = 3 biological replicates. **o** The level of mCG after blastoids transplantation from day 5 to day 11. n = 3 biological replicates. **p-q** Representative bright field images of M-nt-ESCs-derived blastoid (p) and M-iPSCs-derived blastoid (q) developing *in vitro* on day 3. Scale bars, 200 µm. **r** Representative immunofluorescent staining images of GATA3/GATA6/SOX2 in M-nt-ESCs-derived blastoid IVC on day 3. Scale bars, 100 µm. **s** Representative immunofluorescent staining images of GATA3/GATA6/SOX2 in M-iPSCs-derived blastoid IVC on day 3. Scale bars, 100 µm.

Supplementary Fig. 7

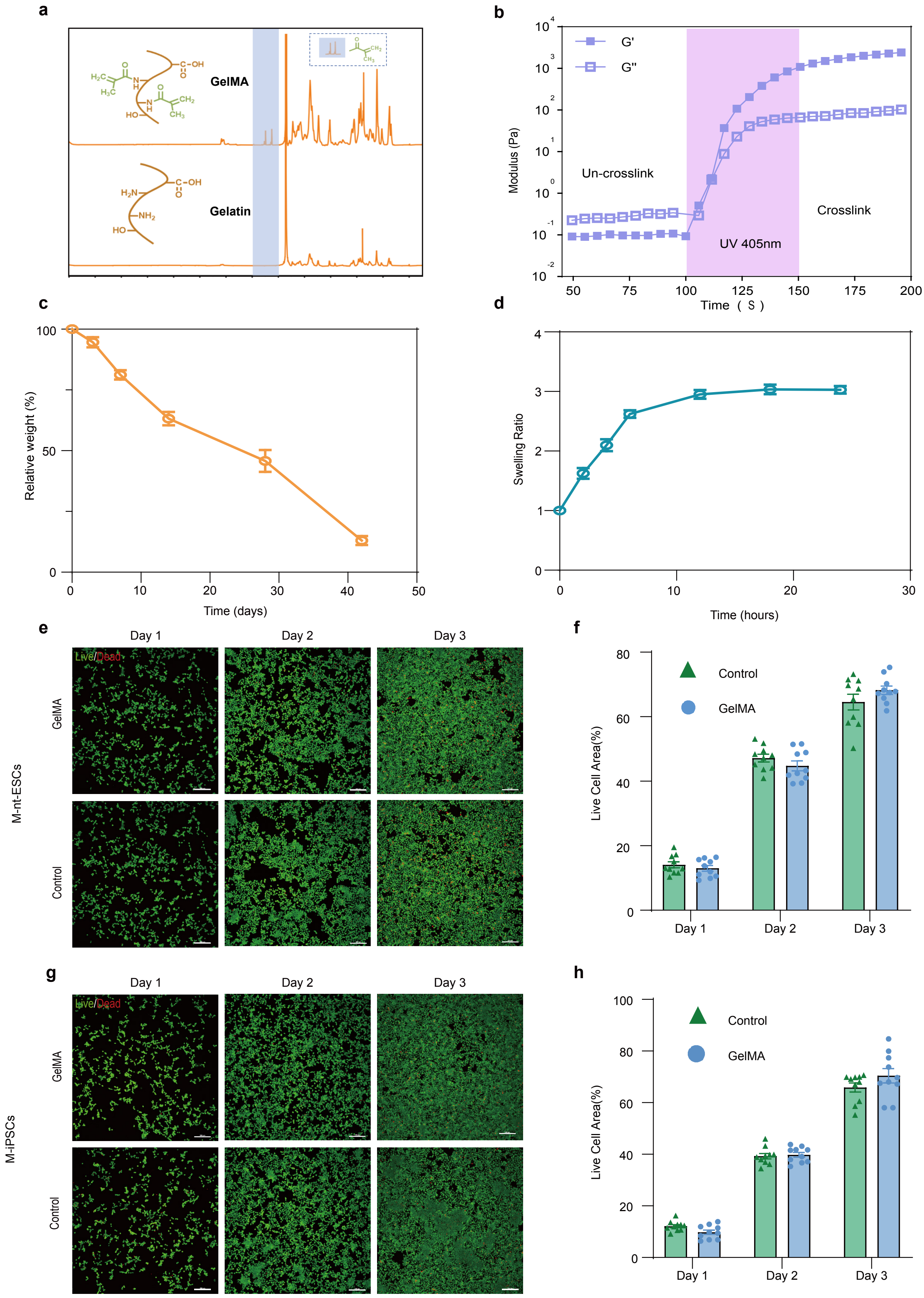

Supplementary Fig.7. Performance of UV Curable GelMA hydrogel.

a ^{1}H-NMR spectra of GelMA and Gelatin. **b** UV curing process and final storage modulus of hydrogel. Oscillation time scanning: strain 1%, angular rate 5 rad/s; Irradiation light source: 405 nm, 30 mW/cm^2, 30 s. **c** The degradation dynamics of GelMA capsules, n = 5 biological replicates. Error bars, mean ± S.E.M. **d** The swelling ratio of GelMA capsules. n = 5 replicates. Error bars, mean ± S.E.M. **e** Live/dead staining images of M-nt-ESCs cultured on GelMA and control groups for 1, 2, and 3 days. Scale bars, 200 μm. **f** Viable cell area obtained from the live/dead staining assay of supplementary fig.7e. n =10 biological replicates. Error bars, mean ± S.E.M. **g** Live/dead staining images of M-iPSCs cultured on GelMA and control groups for 1, 2, and 3 days. Scale bars, 200 μm. **h** Viable cell area obtained from the live/dead staining assay of supplementary fig.7g. n = 10 biological replicates. Error bars, mean ± S.E.M.

Supplementary Fig. 8

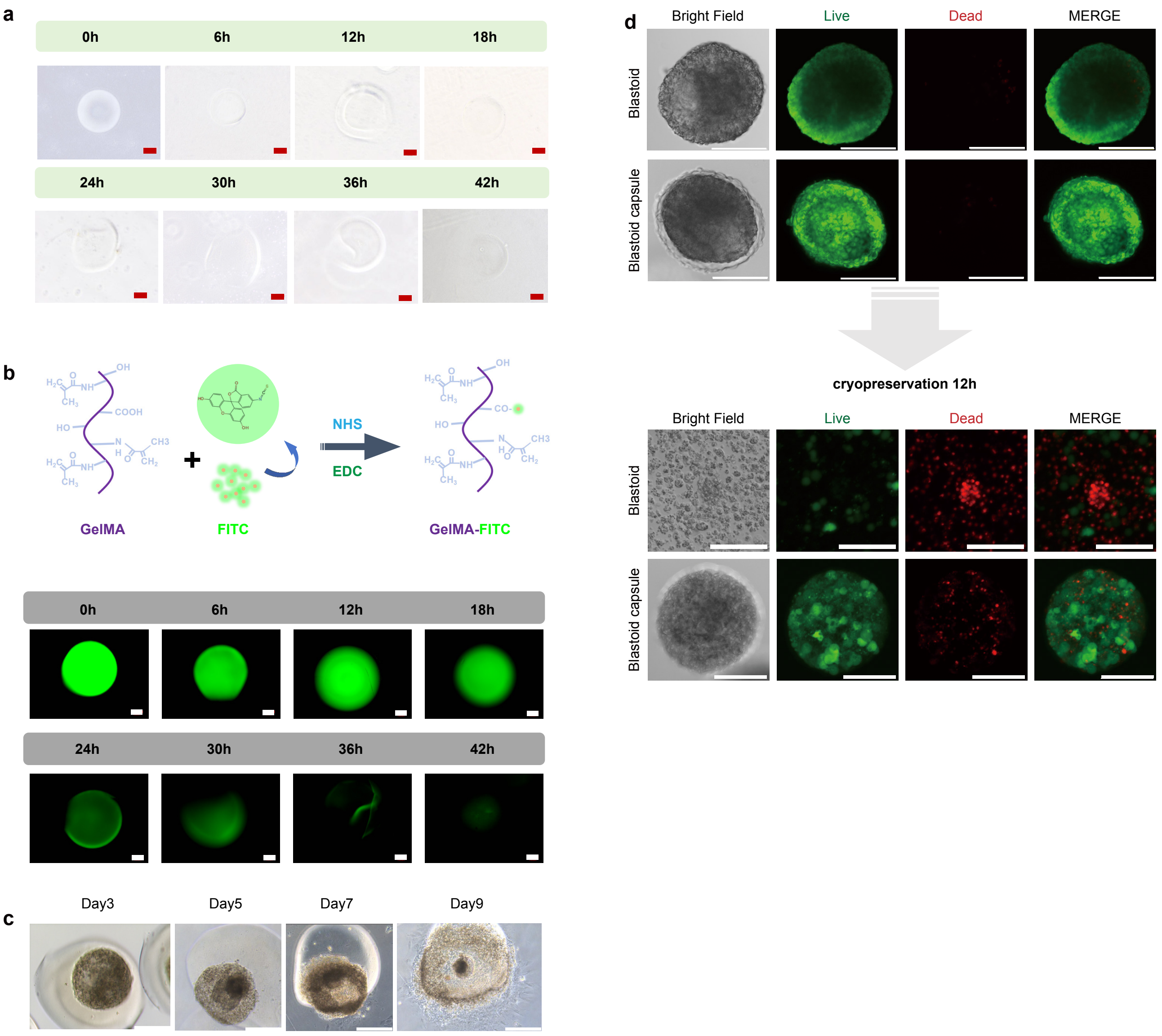

Supplementary Fig.8. Degradation of hydrogel, and blastoid growth, delivery and cryopreservation in blastoid capsule.

a Biodegradation of microspheres with a diameter of 300 µm prepared using high-throughput microfluidic devices during 42 hours. **b** *In vitro* degradation experiment of GelMA-FITC hydrogel. Scale bars, 100 µm. **c** The blastoids gradually grow as the hydrogel degrades *in vitro*. Scale bars, 155.5 µm. **d** Representative morphology and immunofluorescence staining images of live (green) / dead (red) cells of the blastoids and blastoid capsules after 12 hours of cryopreservation. Scale bars, 100 µm.
