# Supplementary material for: Highly efficient construction of monkey blastoid capsules from aged somatic cells
Source: Nat Commun. 2025 Jan 28;16:1130. doi: 10.1038/s41467-025-56447-z (PMC11775175; doi:10.1038/s41467-025-56447-z)
Supplement: Supplementary file 2 — Description of Additional Supplementary Files [file 41467_2025_56447_MOESM2_ESM.pdf]

Description of Additional Supplementary Files

Supplementary Data 1 - The minimum dataset necessary to interpret, verify, and extend the research findings presented in this manuscript is available without restriction. The data have been deposited in a public repository.The data are accessible through accession number PRJCA030207 and are publicly accessible at https ://ngdc.cncb.ac.cn/gsa., which is a recommended repository as per the Springer Nature research data policy. The top 80 genes of ELC, TLC, and HLC in blastoids are listed in Supplementary Data 1

Supplementary Data 2 - The GO and KEGG analysis results of the top 80 genes of ELC, TLC, and HLC in blastoids are listed in Supplementary Data 2

Supplementary Movie 1: The production capacity reaches 4000 blastoid capsules per hour, enabling scalable production of blastoid capsules.
