# Supplementary material for: Highly efficient construction of monkey blastoid capsules from aged somatic cells
Source: Nat Commun. 2025 Jan 28;16:1130. doi: 10.1038/s41467-025-56447-z (PMC11775175; doi:10.1038/s41467-025-56447-z)
Supplement: Supplementary file 6 — Reporting Summary [file 41467_2025_56447_MOESM6_ESM.pdf]

nature portfolio

Corresponding author(s): Yuyu Niu

Last updated by author(s): Jan 9, 2025

Reporting Summary

Nature Portfolio wishes to improve the reproducibility of the work that we publish. This form provides structure for consistency and transparency in reporting. For further information on Nature Portfolio policies, see our Editorial Policies and the Editorial Policy Checklist.

Statistics

For all statistical analyses, confirm that the following items are present in the figure legend, table legend, main text, or Methods section.

n/a	Confirmed	
☐	☒	The exact sample size (*n*) for each experimental group/condition, given as a discrete number and unit of measurement
☐	☒	A statement on whether measurements were taken from distinct samples or whether the same sample was measured repeatedly
☐	☒	The statistical test(s) used AND whether they are one- or two-sided<br>*Only common tests should be described solely by name; describe more complex techniques in the Methods section.*
☒	☐	A description of all covariates tested
☒	☐	A description of any assumptions or corrections, such as tests of normality and adjustment for multiple comparisons
☐	☒	A full description of the statistical parameters including central tendency (e.g. means) or other basic estimates (e.g. regression coefficient) AND variation (e.g. standard deviation) or associated estimates of uncertainty (e.g. confidence intervals)
☐	☒	For null hypothesis testing, the test statistic (e.g. *F*, *t*, *r*) with confidence intervals, effect sizes, degrees of freedom and *P* value noted<br>*Give P values as exact values whenever suitable.*
☒	☐	For Bayesian analysis, information on the choice of priors and Markov chain Monte Carlo settings
☐	☒	For hierarchical and complex designs, identification of the appropriate level for tests and full reporting of outcomes
☒	☐	Estimates of effect sizes (e.g. Cohen's *d*, Pearson's *r*), indicating how they were calculated

Our web collection on statistics for biologists contains articles on many of the points above.

Software and code

Policy information about availability of computer code

Data collection

Cultured cell images were taken by Leica. Confocal images were taken by AX RConfocal Microscope (Nikon) or Leica TCS SP8 confocal microscope (Leica). The mCG were analyzed by electrochemiluminescence on a Roche Cobas e411.

RNA-seq data sets were processed and analyzed using the following tools:
Seurat (v4.1.1)
R (v4.1.2)
DoubletFinder (v2.0.3)
CellRanger (v.7.0.0)

Code used in this project is provided at https://figshare.com/articles/dataset/_b_Highly_Efficient_b_b_Construction_of_Monkey_b_b_Blastoid_Capsules_from_Aged_Somatic_Cells_b_/27020290

Data analysis	Statistic analysis and figure were draw by Prism 10 (v10.3.0) or Microsoft Excel (v16.92). Immunofluorescence staining images were adjusted by image J (1.53k).<br><br>RNA-seq data sets were processed and analyzed using the following tools:<br>Seurat (v4.1.1)<br>R (v4.1.2)<br>DoubletFinder (v2.0.3)<br>CellRanger (v.7.0.0)

For manuscripts utilizing custom algorithms or software that are central to the research but not yet described in published literature, software must be made available to editors and reviewers. We strongly encourage code deposition in a community repository (e.g. GitHub). See the Nature Portfolio guidelines for submitting code & software for further information.

Data

Policy information about availability of data

All manuscripts must include a data availability statement. This statement should provide the following information, where applicable:

- Accession codes, unique identifiers, or web links for publicly available datasets
- A description of any restrictions on data availability
- For clinical datasets or third party data, please ensure that the statement adheres to our policy

The main data supporting the results in this study are available within the paper and its Supplementary Information. The sequence data reported in this paper have been deposited in the Genome Sequence Archive in the National Genomics Data Center, Chinese Academy of Sciences, under accession number PRJCA030207 [https://ngdc.cncb.ac.cn/gsa/browse/CRA019019/CRX1191842] and are publicly accessible at https://ngdc.cncb.ac.cn/gsa. Other data used in this manuscript: Natural monkey embryo data (GSE148683 [https://www.ncbi.nlm.nih.gov/geo/query/acc.cgi?acc=GSE148683], GSE74767 [https://www.ncbi.nlm.nih.gov/geo/query/acc.cgi?acc=GSE74767] and GSE75764 [https://www.ncbi.nlm.nih.gov/geo/query/acc.cgi?acc=GSE75764]); monkey blastoid data (GSE218375 [https://www.ncbi.nlm.nih.gov/geo/query/acc.cgi?acc=GSE218375]); amniotic ectoderm-like cells data (GSE134571 [https://www.ncbi.nlm.nih.gov/geo/query/acc.cgi?acc=GSE134571]); human blastoid data (GSE171820 [https://www.ncbi.nlm.nih.gov/geo/query/acc.cgi?acc=GSE171820]). Every request about data availability can be directed to, and will be fulfilled by, the corresponding author.

Research involving human participants, their data, or biological material

Policy information about studies with human participants or human data. See also policy information about sex, gender (identity/presentation), and sexual orientation and race, ethnicity and racism.

Reporting on sex and gender	Not applicable
Reporting on race, ethnicity, or other socially relevant groupings	Not applicable
Population characteristics	Not applicable
Recruitment	Not applicable
Ethics oversight	Not applicable

Note that full information on the approval of the study protocol must also be provided in the manuscript.

Field-specific reporting

Please select the one below that is the best fit for your research. If you are not sure, read the appropriate sections before making your selection.

☒ Life sciences ☐ Behavioural & social sciences ☐ Ecological, evolutionary & environmental sciences

For a reference copy of the document with all sections, see nature.com/documents/nr-reporting-summary-flat.pdf

Life sciences study design

All studies must disclose on these points even when the disclosure is negative.

Sample size	No statistical methods were used to predetermine sample size. For each experiment, the sample size is determined based on an interval of confidence equal or above 95% as described in the methods and in the text, and on the sampling error, which is estimated based on the standard deviations during pilot studies.
Data exclusions	The filtered matrixes from CellRanger output were imported into Seurat (v4.1.1) in R (v4.1.2) for quality control and further analysis. Cells with low quality (< 2000 genes/cell, > 7500 genes/cell, < 3000 UMI,> 40000 UMI and > 10% mitochondrial genes) were excluded.
Replication	All attempts at replication were successful over 3 independent experiments.

Randomization	To generate blastoids, monkey PSCs were counted and seeded into Aggrewell, and randomly allocated into each microwell. All generated blastoid were randomly picked and used for the following experiments: cell number counting, width and length measurements, immunofluorescence analysis, stem cell lines derivation, chemical inhibitor treatments and prolonged culture in IVC media. For experiments other than those mentioned here, random allocation is not relevant. For example, for stem cell differentiation experiments the investigators need to know specific stem cell type, plating density, differentiation protocol and differentiation time and therefore random allocation was not performed for these experiments.
Blinding	Data collection and analysis (e.g. immunofluoresence analysis, cell number counting, blastoids width and length measurements) were blinded.

Reporting for specific materials, systems and methods

We require information from authors about some types of materials, experimental systems and methods used in many studies. Here, indicate whether each material, system or method listed is relevant to your study. If you are not sure if a list item applies to your research, read the appropriate section before selecting a response.

Materials & experimental systems

n/a	Involved in the study
☐	☒ Antibodies
☐	☒ Eukaryotic cell lines
☒	☐ Palaeontology and archaeology
☐	☒ Animals and other organisms
☒	☐ Clinical data
☒	☐ Dual use research of concern
☒	☐ Plants

Methods

n/a	Involved in the study
☒	☐ ChIP-seq
☐	☒ Flow cytometry
☒	☐ MRI-based neuroimaging

Antibodies

Antibodies used

anti-OCT4-M(Santa Cruz Biotechnology #sc-5279, dilution 1:500)
anti-OCT4-G(R&D systems #AF1759, dilution 1:500)
anti-OCT4-R(Abcam #ab181557, dilution 1:2000)
anti-NANOG-G(R&D systems #AF1997, dilution 1:1000)
anti-SOX2-R(Abcam #ab93689, dilution 1:1000)
anti-SOX2-M(Santa Cruz Biotechnology #sc-365823, dilution 1:500)
anti-GATA3-R(Cell signaling technology #5852S, dilution 1:2000)
anti-GATA3-M(Santa Cruz Biotechnology #sc-268, dilution 1:500)
anti-GATA4-M(Santa Cruz Biotechnology #sc-25310, dilution 1:500)
anti-GATA4-G(R&D systems #BAF2606, dilution 1:1000)
anti-GATA6-G(R&D systems #AF1700, dilution 1:1000)
anti-ZO-1(Invitrogen #33-9100, dilution 1:500)
anti-TFAP2C(Santa Cruz Biotechnology #sc-12762, dilution 1:500)
anti-CDX2-R(Abcam #ab195007, dilution 1:1000)
anti-CDX2-M(Abcam #ab76541, dilution 1:1000)
anti-SOX17(R&D systems #AF1924, dilution 1:500)
anti-HLA-G(Proteintech #66447-1-Ig, dilution 1:500)
anti-SDC1-M(abcam # ab34164, dilution 1:1000)
anti-T(Cell signaling technology #81694, dilution 1:2000)
anti-CCR7-R(Invitrogen #MA5-31992, dilution 1:1000)
anti-NESTIN-H(Stemcell #60091.1, dilution 1:1000)
anti-FOXA2-M(Abcam #ab60721, dilution 1:1000)
anti-αSMA-M(Abcam #ab7817, dilution 1:1000)
anti-TUJ1-M(Abcam #ab78078, dilution 1:1000)
anti-GATA2-R(Abcam #ab109241, dilution 1:2000)
DAPI(Roche #10236276001, dilution 1:5000)
Donkey anti-Rabbit 488(Invitrogen #A-21206, dilution 1:5000)
Donkey anti-Rabbit 555(Invitrogen #A-31572, dilution 1:5000)
Donkey anti-Rabbit 647(Abcam #ab150063, dilution 1:5000)
Donkey anti-Mouse 488(Invitrogen #A-21202, dilution 1:2000)
Donkey anti-Mouse 555(Invitrogen #A-31570, dilution 1:2000)
Donkey anti-Goat 488(Invitrogen #A-11055, dilution 1:5000)
Donkey anti-Goat 647(Abcam #ab150135, dilution 1:5000)

Validation

anti-CCR7(https://www.thermofisher.cn/cn/zh/antibody/product/CCR7-Antibody-clone-SR36-04-Recombinant-Monoclonal/MA5-31992)
anti-NESTIN(https://www.stemcell.com/products/anti-human-nestin-antibody-clone-10c2.html)
anti-FOXA2(https://www.abcam.cn/products/primary-antibodies/foxa2-antibody-7e6-bsa-and-azide-free-ab60721.html)
anti-αSMA(https://www.abcam.cn/products/primary-antibodies/alpha-smooth-muscle-actin-antibody-1a4-ab7817.html)
anti-TUJ1(https://www.abcam.cn/products/primary-antibodies/beta-iii-tubulin-antibody-2g10-neuronal-marker-ab78078.html)
anti-GATA2(https://www.abcam.cn/products/primary-antibodies/gata2-antibody-epr28222-ab109241.html)

anti-OCT4(https://www.scbt.com/zh/p/oct-3-4-antibody-c-10)
anti-OCT4(https://www.rndsystems.com/cn/products/human-mouse-oct-3-4-antibody_af1759)
anti-OCT4(https://www.abcam.cn/products/primary-antibodies/oct4-antibody-epr17929-chip-grade-ab181557.html)
anti-NANOG(https://www.rndsystems.com/cn/products/human-nanog-antibody_af1997)
anti-SOX2-R(https://www.abcam.cn/products/primary-antibodies/sox2-antibody-sp76-ab93689.html)
anti-SOX2-M(https://www.scbio.cn/zh/p/sox-2-antibody-e-4)
anti-GATA3(https://www.cellsignal.cn/products/primary-antibodies/gata-3-d13c9-xp-rabbit-mab/5852)
anti-GATA3(https://www.scbio.cn/zh/p/gata-3-antibody-hg3-31)
anti-GATA4-M(https://www.scbio.cn/p/gata-4-antibody-g-4)
anti-GATA4-G(https://www.rndsystems.com/cn/products/human-gata-4-biotinylated-antibody_baf2606)
anti-GATA6(https://www.rndsystems.com/cn/products/human-gata-6-antibody_af1700)
anti-ZO-1(https://www.thermofisher.cn/cn/zh/antibody/product/ZO-1-Antibody-clone-ZO1-1A12-Monoclonal/33-9100)
anti-TFAP2C(https://www.scbio.cn/zh/p/ap-2gamma-antibody-6e4-4)
anti-CDX2(https://www.abcam.cn/products/primary-antibodies/alexa-fluor-488-cdx2-antibody-epr2764y-ab195007.html)
anti-CDX2-M(https://www.abcam.cn/products/primary-antibodies/cdx2-antibody-epr2764y-ab76541.html)
anti-SOX17(https://www.rndsystems.com/cn/products/human-sox17-antibody_af1924)
anti-HLA-G(https://www.ptgcn.com/products/HLA-G-Antibody-66447-1-Ig.htm)
anti-SDC1-M(https://www.abcam.cn/products/primary-antibodies/syndecan-1-antibody-b-a38-ab34164.html)
anti-T(https://www.cellsignal.cn/products/primary-antibodies/brachyury-d2z3j-rabbit-mab/81694)
DAPI(https://www.sigmaaldrich.cn/CN/zh/product/roche/10236276001)
Donkeyanti-Rabbit488(https://www.thermofisher.cn/cn/zh/antibody/product/Donkey-anti-Rabbit-IgG-H-L-Highly-Cross-Adsorbed-Secondary-Antibody-Polyclonal/A-21206)
Donkeyanti-Rabbit555(https://www.thermofisher.cn/cn/zh/antibody/product/Donkey-anti-Rabbit-IgG-H-L-Highly-Cross-Adsorbed-Secondary-Antibody-Polyclonal/A-31572)
Donkeyanti-Rabbit647(https://www.abcam.cn/products/secondary-antibodies/donkey-rabbit-igg-hl-alexa-fluor-647-preadsorbed-ab150063.html)
Donkeyanti-Mouse488(https://www.thermofisher.cn/cn/zh/antibody/product/Donkey-anti-Mouse-IgG-H-L-Highly-Cross-Adsorbed-Secondary-Antibody-Polyclonal/A-21202)
Donkeyanti-Mouse555(https://www.thermofisher.cn/cn/zh/antibody/product/Donkey-anti-Mouse-IgG-H-L-Highly-Cross-Adsorbed-Secondary-Antibody-Polyclonal/A-31570)
Donkeyanti-Goat488(https://www.thermofisher.cn/cn/zh/antibody/product/Donkey-anti-Goat-IgG-H-L-Cross-Adsorbed-Secondary-Antibody-Polyclonal/A-11055)
Donkeyanti-Goat647(https://www.abcam.cn/products/secondary-antibodies/donkey-goat-igg-hl-alexa-fluor-647-preadsorbed-ab150135.html)

Eukaryotic cell lines

Policy information about cell lines and Sex and Gender in Research

Cell line source(s)	Monkey ESCs were derived from blastocyst, which were reported previously (Wu et al., Protein&Cell, 2024). Monkey dermal fibroblasts were derived from tissues of cynomolgus monkeys for reprogramming experiments and SCNT experiments.<br>Fibroblast, M-nt-ESC and M-iPSCs:<br>The Fibroblast-312 and the Fibroblast-050, were derived from female cynomolgus monkeys aged 15 years old. The Fibroblast-418 and the Fibroblast-058, were derived from female rhesus monkeys aged 22 years old. The Fibroblast-372 was derived from female rhesus monkeys aged 25 years old. The Fibroblast-182004 and the Fibroblast-040, were derived from female cynomolgus monkeys aged 1 years old. The Fibroblast-004, the Fibroblast-006 and the Fibroblast-446 were derived from female rhesus monkeys aged 5 years old. In this study, M-iPSCs were derived from Fibroblast-312, Fibroblast-050, Fibroblast-182004 and Fibroblast-040; M-nt-ESCs were derived from Fibroblast-418, Fibroblast-058, Fibroblast-372, Fibroblast-004, Fibroblast-006, Fibroblast-446. In the experiments, none of the monkey underwent genetic modifications such as gene editing.<br>M-ESC:<br>XF-ESC-1, XF-ESC-2, XF-ESC-3 CES1_1-XF PSC and CES_N from Wu et al.'s report
Authentication	All monkey PSCs include ESCs, iPSCs and nt-ESCs were validated by immunostaining, karyotype analysis and teratoma assay.
Mycoplasma contamination	Cells were routinely tested for mycoplasma contamination. No contamination was detected weekly.
Commonly misidentified lines<br>(See ICLAC register)	No commonly misidentified cell lines were used in the study.

Animals and other research organisms

Policy information about studies involving animals; ARRIVE guidelines recommended for reporting animal research, and Sex and Gender in Research

Laboratory animals	Healthy male and female cynomolgus monkeys and rhesus monkeys, ranging in age from 1 to 25 years, were selected for use in this study. The cynomolgus monkeys and rhesus monkeys were housed with a 12-h light/dark cycle between 06:00 and 18:00 in a temperature-controlled room (22°C ± 1°C) with free access to water and food. The mouse were housed with a 12-hour light/dark cycle between 6:00 and 18:00 in a temperature-controlled room (22 ± 1 °C) with free access to water and food.
Wild animals	No wild animals were used.
Reporting on sex	This study did not involve subjects or materials that require sex-based differentiation.

Field-collected samples	No field-collected samples were used.
Ethics oversight	All animals were housed at the State Key Laboratory of Primate Biomedical Research (LPBR). Every experiment involving animals have been carried out following a protocol approved by an ethical commission. All animal and experiment procedures were approved by the ethical committee of the LPBR and performed by following the guidelines of the Association for Assessment and Accreditation of Laboratory Animal Care International (AAALAC) for the ethical treatment of NHPs. All animal procedures were approved in advance by State Key Laboratory of Primate Biomedical Research (no. LPBR202104016 and PZWH (YN) K2023-0022).

Note that full information on the approval of the study protocol must also be provided in the manuscript.

Plants

Seed stocks	Not applicable
Novel plant genotypes	Not applicable
Authentication	Not applicable

Flow Cytometry

Plots

Confirm that:

☒ The axis labels state the marker and fluorochrome used (e.g. CD4-FITC).

☒ The axis scales are clearly visible. Include numbers along axes only for bottom left plot of group (a 'group' is an analysis of identical markers).

☒ All plots are contour plots with outliers or pseudocolor plots.

☒ A numerical value for number of cells or percentage (with statistics) is provided.

Methodology

Sample preparation	Blastoids were dissociated using Accutase(Gibco), washed twice with DPBS and filtered through a Falcon® 40 µm Cell Strainer (Falcon, 352340). Cells were suspended with PBS. They were loaded into BD LSR Fortessa.
Instrument	BD LSR Fortessa
Software	Analyzed by Flowjo
Cell population abundance	Sample purity was verified as necessary.
Gating strategy	Preliminary FSC/SCC gates were used to remove debris, doublets and other aggregated particles from the cell population. Boundaries between positive and negative staining cell populations are defined by using unstained negative control.

☐ Tick this box to confirm that a figure exemplifying the gating strategy is provided in the Supplementary Information.
